# Heat Shock Proteins, a Double-Edged Sword: Significance in Cancer Progression, Chemotherapy Resistance and Novel Therapeutic Perspectives

**DOI:** 10.3390/cancers16081500

**Published:** 2024-04-14

**Authors:** Dominika Kunachowicz, Magdalena Król-Kulikowska, Wiktoria Raczycka, Jakub Sleziak, Marta Błażejewska, Julita Kulbacka

**Affiliations:** 1 Department of Pharmaceutical Biochemistry, Faculty of Pharmacy, Wroclaw Medical University, Borowska 211A, 50-556 Wroclaw, Poland; dominika.kunachowicz@student.umw.edu.pl (D.K.); magdalena.krol-kulikowska@umw.edu.pl (M.K.-K.); 2 Faculty of Medicine, Wroclaw Medical University, Pasteura 1, 50-367 Wroclaw, Poland; wiktoria.raczycka@student.umw.edu.pl (W.R.); jakub.sleziak@student.umw.edu.pl (J.S.); marta.blazejewska@student.umw.edu.pl (M.B.); 3 Department of Molecular and Cellular Biology, Faculty of Pharmacy, Wroclaw Medical University, Borowska 211A, 50-556 Wroclaw, Poland; 4 Department of Immunology and Bioelectrochemistry, State Research Institute Centre for Innovative Medicine Santariškių g. 5, LT-08406 Vilnius, Lithuania; 5 DIVE IN AI, 53-307 Wroclaw, Poland

**Keywords:** heat shock proteins, cancer progression, chemotherapy resistance, anticancer therapy

## Abstract

**Simple Summary:**

Among the many mechanisms developed by cancer cells in order to survive and sustain constant proliferation under stress conditions, a system based on heat shock proteins (Hsps) has attracted a lot of scientific attention. On the one hand, the activity of these proteins enables cancer cells to endure chemotherapy, while, on the other, this fact can be used in the development of novel strategies of cancer treatment based on Hsp inhibition. This review article offers a comprehensive insight into complex roles of Hsps in cancer progression, with particular regard to drug resistance development, and presents recent advances in anticancer therapy interfering with their function.

**Abstract:**

Heat shock proteins (Hsps) are involved in one of the adaptive mechanisms protecting cells against environmental and metabolic stress. Moreover, the large role of these proteins in the carcinogenesis process, as well as in chemoresistance, was noticed. This review aims to draw attention to the possibilities of using Hsps in developing new cancer therapy methods, as well as to indicate directions for future research on this topic. In order to discuss this matter, a thorough review of the latest scientific literature was carried out, taking into account the importance of selected proteins from the Hsp family, including Hsp27, Hsp40, Hsp60, Hsp70, Hsp90 and Hsp110. One of the more characteristic features of all Hsps is that they play a multifaceted role in cancer progression, which makes them an obvious target for modern anticancer therapy. Some researchers emphasize the importance of directly inhibiting the action of these proteins. In turn, others point to their possible use in the design of cancer vaccines, which would work by inducing an immune response in various types of cancer. Due to these possibilities, it is believed that the use of Hsps may contribute to the progress of oncoimmunology, and thus help in the development of modern anticancer therapies, which would be characterized by higher effectiveness and lower toxicity to the patients.

## 1. Introduction

In a constantly changing environment, single cells as well as whole organisms are exposed to environmental, metabolic and pathophysiological stresses. This state requires the existence of certain adaptive mechanisms that will provide a counterbalance to this type of phenomenon. One such system is organized by heat shock proteins (Hsps). This family of proteins plays an important role in maintaining cellular homeostasis and protecting cells from various stressors, such as heat, infections and toxins [1,2]. Other functions of Hsps include participation in the correct folding of proteins and maintaining their stability, as well as their assembly, translocation and degradation in cooperation with the ubiquitin–proteasome system, both associated with regular processes of cell biology and exposure to stresses. Since proteins are involved in basically all processes governing the functionality of cells, the maintenance of their proper three-dimensional native structure is of utmost importance in a biological context and critical for their physiological function. Notably, proteins are most sensitive to various cellular stresses among biomolecules [3,4]. Therefore, maintaining the integrity of the cellular proteome—called proteostasis—requires the presence and activity of specialized agents, which indeed are Hsps, among others. Their crucial role in protein quality control and regulation is indicated by the fact that Hsps are highly conserved in prokaryotes and eukaryotes throughout evolution [5]. Hsps interact with exposed hydrophobic regions of unfolded or misfolded proteins to prevent aggregation, and due to such mechanisms of action, they are called “molecular chaperones” [6,7]. Dysregulation of Hsps has been implicated in various diseases, including cancer [8]. However, it should be noted that the role of Hsps in the pathogenesis of cancer is complex. These proteins can perform both protective and procancer functions, depending on the specific member of the Hsp family, the type of cancer and the stage of disease development [9]. In some cases, cancer cells overexpress certain Hsps to promote their survival and resistance to therapies [10]. Special cases here are Hsp70 and Hsp90 proteins, which are often overexpressed in cancer cells and additionally induce the growth and development of these cells, as well as prevent apoptosis [11]. Hsps may also facilitate the metastatic spread of the tumor by promoting cell motility and their adherence to distant organs [12]. The increased activity of Hsps in cancer patients results in an increased concentration of these proteins in biological samples. Hence, these proteins can be used as diagnostic or prognostic markers [13,14,15]. Hsps are also associated with resistance to cancer therapies [16]. It is speculated that their chaperone function may protect cancer cells against the harmful effects of these therapies; therefore, the inhibition of the proteins of the Hsp family could sensitize cancer cells to treatment [17]. The protection of cancer cells in the case of chemotherapy is possible due to the similar functions that Hsps perform in healthy cells. Hsp27, Hsp70 and Hsp90 stabilize many client proteins involved in cell survival and anti-apoptotic pathways. These client proteins can include kinases, transcription factors and apoptotic regulators. By chaperoning these proteins, Hsps prevent their degradation and maintain their activity, allowing cancer cells to survive chemotherapy-induced stress [18,19,20]. What is more, Hsps can reduce the effectiveness of chemotherapy-induced DNA damage and facilitate the repair of such damage [21]. They can also regulate the expression and activity of drug efflux pumps, such as P-glycoprotein (P-gp). These pumps actively transport chemotherapeutics out of cancer cells, reducing their intracellular concentration and effectiveness. By stabilizing or upregulating these pumps, Hsps contribute to drug resistance [22,23].

Such various functions make Hsps potentially attractive targets for research into introducing more efficient anticancer therapies. Clinical trials of small-molecule inhibitors targeting Hsps, which are seen as potential anticancer agents, are of great significance [24,25]. In addition, a better understanding of the signaling pathways in which Hsps are involved may help identify new therapeutic targets to overcome chemoresistance [26,27,28]. Due to the fact that the action of Hsp inhibitors in overcoming chemoresistance is still an area of research, and their effectiveness may vary depending on the type and stage of cancer, taking up the topic in this field seems to be very important for cancer patients. This review focuses on summarizing the role of Hsps in the development of cancer and chemoresistance, taking into account the latest research. Moreover, it also contains a discussion of how targeting Hsps can be used in the development of novel treatments for cancer. This article offers insight into future research directions on Hsps and identifies areas for further research and potential therapeutic development pathways.

## 2. General Features of Hsps

The classification of Hsps is based on their molecular weight. In general, Hsps are divided into the following two main groups: ATP-dependent Hsps of a high molecular mass, such as Hsp40, 60, 70, 90 and 110, and small Hsps (sHsps), consisting of ten members with a major role, played by Hsp27, Hsp20 and αB-crystallin. 

### 2.1. Small Hsps: Classification, Structure–Function Relationship and Activities 

Small Hsps, the molecular weight of which ranges between 12 and 43 kDa, can be further divided into class I, characterized by ubiquitous expression in various cell and tissue types, and tissue-restricted class II. The former is generally associated with stress-induced cell survival mechanisms, while the latter is overall involved in developmental processes, differentiation and tissue-specific functions [3,29,30].

Small Hsps share a common domain structural architecture. Their N-terminal domain (NTD), which is highly variable and divergent in length and sequence, except for a few conserved stretches, presents a disordered structure and contains multiple hydrophobic residues, along with many sites available for phosphorylation. The C-terminal domain (CTD) is rich in polar and charged amino acid residues [31]. What is unique for sHsps, and regarded as their important hallmark, is that these two domains are connected by a flexible structure referred to as the α-crystallin domain (ACD), which is characteristic for all proteins belonging to this family and present regardless of their exact origin and nature. The ACD consists of 80–100 amino acid residues, forming six or seven β-strands organized in two β-sheets [32,33], and is enriched in histidine residues, which participate in the modulation of sHsp activity via enabling the responsiveness to pH changes and metal ions [31]. Meanwhile, CTD and NTD play a key role in the oligomerization and stabilization of the structures formed by sHsps and ensure accessibility for client proteins to interact with [34,35].

Human sHsps present a high degree of structural variations, which can be dimers, heterotetramers and polydisperse co-assembling oligomers, characterized by dynamic behavior due to their ability to exchange monomeric subunits under stress conditions [33]. This feature of sHsps enables the proper recognition of client proteins depending on specific cellular conditions. The oligomerization state of sHsps, regulated by disordered CTD and folded ACD, affects the affinity for a particular substrate, which, therefore, can vary depending on the currently exposed binding interfaces of the sHsps. This intrinsic structural plasticity of sHsps, concerning the ability to expose different binding sites, enables them to bind a wide range of structurally unrelated protein clients [36]. Numerous factors can affect the oligomeric structures of sHsps, including reversible post-translational modifications such as phosphorylation. Moreover, it has been suggested that phosphorylation at different sites can have a distinct influence on the oligomer composition and, consequently, function [37].

Small Hsps, in contrast to high-molecular-weight Hsp family members, do not require ATP to perform their function as protein chaperones. Since sHsps are not actively involved in the folding processes, they are described as holdase chaperones, which, under stress conditions, bind to misfolded or unfolded proteins and thereby prevent them either from forming dysfunctional aggregates with other unstable proteins or from proteolytic degradation [37,38]. According to the most acknowledged model of sHsp action, the chaperone dimers associate with target proteins, while larger oligomers serve as reservoirs of these small species. The discrimination between folded and disordered proteins by sHsps is possible due to the conformational stability and hydrophobicity of the precursor protein intermediates—interactions of high affinity are therefore possible only with highly destabilized structures [39]. Hence, sHsps shield their client proteins and hold them in a folding-competent state to be afterward refolded or disaggregated, which can only be performed by other ATP-dependent chaperones, mainly Hsp40/Hsp70 [40,41,42]. Significantly, sHsps interact not only with proteins prone to aggregation in monomeric, oligomeric, prefibrillar or fibrillar forms, but also with a broader range of biomolecules. The best-known group of sHsp-interacting proteins are cytoskeleton components—tubulin, actin, vimentin and desmin—that are of key importance in controlling the cellular and nuclear shape. The functions of other proteins, which are indicated to interact with sHsps, include signal transduction and the processes of transcription and translation, but the extent of their functional dependence on sHsp chaperone activities is yet unknown [33,43]. The activation of specific kinases (e.g., mitogen-activated protein kinases, MAPKs) in cells occurring under stress conditions, leading to the phosphorylation of certain amino acid residues in sHsp molecules, increases its overall negative charge, which affects its structure and subsequently alters interactions with neighboring protein domains. In addition, phosphorylation is often related to the translocation of sHsps to the nucleus, enhancing their protective activity towards proteins in the face of aggregation as a consequence of cellular stress. Also, the transcription of heat shock response genes is upregulated by activated heat shock factor 1 (HSF1), and the pool of newly translated Hsps oligomerize with previously existing ones. This maximizes the possibility of interacting with and protecting intracellular proteins [39].

### 2.2. An Overview of High-Molecular-Weight Hsps and Their Mechanism of Action

Non-small Hsps possess exceptional intrinsic chaperoning properties, enabling them the restoration of native protein structures upon cytotoxic or proteotoxic stress stimuli [44]. Their major functions include the folding of de novo synthesized proteins, the refolding of stress-denatured ones, the guidance of protein translocation across membranes, the enhancement of the proteolytic degradation of unstable or faulty proteins, the dismantling of the oligomeric protein structure and control over regulatory proteins’ biological activities [45]. They are also able to break down harmful protein aggregates [46]. 

Apart from protein homeostasis maintenance, Hsps play a role in suppressing intracellular apoptosis-inducing pathways, largely contributing to cell survival. They have been shown to interfere with both caspase-dependent and independent cell death programs, affect their downstream and upstream effectors as well as be involved in various interactions at the mitochondrial and lysosomal level [47]. Several members of this group have been demonstrated to play a role in innate immunity and antigen cross-presentation [48]. Due to the higher diversity of high-molecular-weight Hsps in comparison with small Hsp family members, their unique features will be provided and discussed in the sections dedicated to specific Hsps further in the manuscript.

Hsps, which are in general intracellular proteins, can be mobilized and translocated to the cell membrane to be released into the surrounding environment, although, due to the lack of a signal peptide in their structure, it does not involve a classical secretion pathway [48]. High-molecular-weight Hsps also present a domain structure, but their exact architecture varies between specific families. Most of these proteins possess a conserved N-terminal nucleotide-binding domain (NBD) with adenosine triphosphatase (ATPase) activity and a C-terminal substrate-binding domain (SBD). The binding of ATP induces alterations in the conformation of the peptide-binding site of the Hsp, which in turn leads to changes in its substrate affinity. When the ATP is bound, the Hsp forms a complex with the unfolded or misfolded protein, enabling proper folding. With the following ATP hydrolysis by the intrinsic ATPase activity of NBD, the Hsp is released from the folded client and can bind another peptide after the next ATP molecule is attached, and a new cycle begins [45]. The exact mechanism of folding newly synthesized proteins or restoring the proper conformation in those that are aggregated or misfolded depends on the specific Hsp structure and cooperation between the Hsps, such as Hsp10, which functions as a co-chaperone for Hsp60, and Hsp40, which is necessary for Hsp70 activity [49]. The assistance from the co-chaperones increases the rate of protein client association and enhances the ATPase activity and nucleotide exchange [19].

### 2.3. Hsps—Major Cancer Chaperones

The protective role of Hsps in proteostasis, crucial in maintaining the functionality of cells, tissues and systems, becomes a concern in tumorigenesis, since the rapid upregulation of Hsp expression and the enhancement of their chaperone activity is characteristic of cancers. Cancer cells, compared to healthy ones, seem to rely on the Hsp chaperone system to a higher extent [10]. According to the accepted paradigm, the malignant transformation of cells is associated with the development of the proteotoxic stress phenotype. Proteome homeostasis is disturbed during tumorigenesis, with several mechanisms conferring this effect. Genetic alterations in cancer cells, such as aneuploidy or gene amplifications, lead to imbalanced protein synthesis, with an accumulation of mutated oncoproteins [50]. Also, the components of the translational machinery are dysregulated, fostering oncogenic effects [51], while oxidative stress conditions in such cells contribute to the intensification of protein damage. In order to counteract such proteotoxic stress and survive, cancer cells activate the multifaceted heat shock response [50]. Oxidative stress also positively influences the activity of Hsps and is regarded as a favorable condition for tumorigenesis and cancer progression, partly due to this chaperone activation [10].

**Figure 1:**
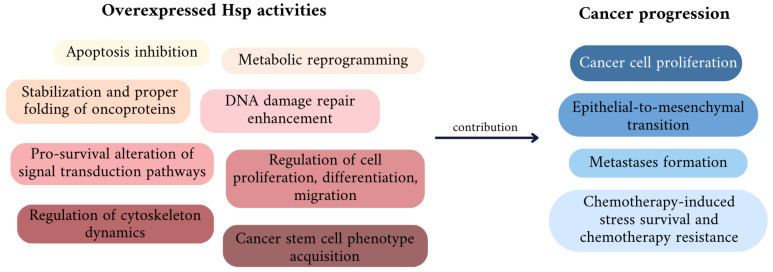
General functions of the heat shock protein family (Hsps) associated with tumorigenesis and cancer progression.

Hsps in cancer cells interact with and stabilize numerous proteins acknowledged as oncogenic, including Bcr-Abl, mutant p53, B-Raf kinase, Akt, cyclin D1, cyclin-dependent kinase 4 (CDK4) and ErbB2/Her2 [52]. The elevated expression and activity of individual Hsps potentiates oncogenic events mediated by these proteins. Moreover, Hsps can affect signal transduction pathways related to cancer cell viability and chemoresistance, enabling their adaptation to stress conditions during chemotherapy and survival. In cancers, Hsps act as chaperones of cell proliferation, enabling the evasion of apoptosis and antigrowth signals. They also contribute to indefinite cell divisions, senescence avoidance and fuel cancer invasion and metastatic processes [19]. In addition, a number of recent reports suggest that Hsps participate in the regulation of DNA repair signaling pathways, which provides the cells an additional adaptation to endure stresses, such as those that are chemotherapy-related, and thereby increases cancer cell survival, promoting chemoresistance [53] (Figure 1). In the following sections corresponding to particular Hsps, their roles in cancer progression and chemoresistance will be evaluated.

## 3. The Role of Particular Hsps in Cancer Development and Drug Resistance

### 3.1. Hsp27

#### 3.1.1. Hsp27 as a Small Hsp Family Member

Human Hsp27 was firstly described in the early 1980s, when it was observed that elevating the temperature of HeLa cell incubation was associated with the appearance of a previously unknown protein with a distinct molecular mass of 27 kDa [32]. Hsp27, belonging to small Hsps (sHsps), is granted particular importance, since it has been demonstrated to be relevant in multiple biological processes, such as development and immunity, as well as diseases [54]. Hsp27 is ubiquitously distributed in human tissues, where it exists in a range of oligomeric states, undergoing concentration-dependent dissociation from polydisperse ensembles of large oligomers to dimers, with frequent subunit exchange between them [55,56]. As established in vitro and in vivo, this phenomenon has a crucial role in modulating Hsp27 chaperone activity. The oligomerization of Hsp27 is dependent on its phosphorylation state, the occurrence of which is determined by conditions in the cell. Large oligomers are created in normal conditions, where Hsp27 then remains unphosphorylated, whereas the stress-induced phosphorylation of Hsp27 leads to the exposure of the N-terminal domain and the dissociation of the oligomers, enabling it to bind with destabilized proteins. The main phosphorylation sites of Hsp27 are serine residues located in the N-terminal domain, and this fact supports the role played by this domain in the stability of oligomers [31,55]. The phosphorylation of Hsp27 causes its dissociation to dimers or tetramers, and is also related to the switching from its chaperone activity into a cell signaling molecule [57].

#### 3.1.2. Involvement in Cancer Progression

Hsp27 is implicated in cancer progression on multiple levels. Although it influences numerous processes related to tumor initiation, invasiveness and metastasis in many mechanisms, as well as its overexpression in various cancer types, and has been linked to more advanced disease stages and a worse prognosis, it is not regarded as a clinically relevant cancer biomarker [58]. The pro-survival and anti-apoptotic activities of Hsp27 are well known, and, what is more, it has also been found to participate in cancer stem cell adaptation to stress conditions and, therefore, increase their viability [59]. Additionally, Hsp27 facilitates cell cycle progression and increases the migratory activity of cancer cells [59], which will be discussed below in more detail.

The molecular bases of Hsp27’s ability to inhibit apoptosis, which plays a crucial role in cancer progression and chemotherapy resistance, have been well investigated to date. Hsp27, via several regulatory mechanisms, contributes to the intrinsic and extrinsic pathway of apoptosis blockage, with a more evident protective effect in the case of the Fas-mediated extrinsic pathway [60,61]. Hsp27 is able to interact with various factors implicated in the intrinsic apoptotic process; on the one hand, this protein, via direct interaction with cytochrome c after its release from the mitochondria, prevents caspase activation and, consequently, caspase-mediated apoptosis; on the other hand, Hsp27 upregulates upstream signaling cascades, such as the phosphatidylinositol-3-kinase (PI3K) pathway [62]. Activated PI3K further phosphorylates inositol, present in the plasma membrane, attracting the serine/threonine Akt kinase, which affects multiple components of the apoptotic machinery, e.g., inhibits the mitochondrial translocation of Bax, which is responsible for preventing pore formation in the outer mitochondrial membrane, and the release of cytochrome c. It has been observed that Hsp27 can bind to Akt, which is crucial for its activation in stressed cells, and directly interacts with Bax, ultimately suppressing apoptosis [62,63]. Regarding the intrinsic pathway, Hsp27 also exerts anti-apoptotic effects by directly interacting with the caspase-3 prodomain, thereby inhibiting its proteolytic activation, hindering apoptosome formation via binding to cytochrome c and preventing the release of Smac from the mitochondria [64]. Phosphorylated Hsp27 can also inhibit Fas-mediated apoptosis as a result of its interaction with the death domain-associated protein (Daxx), which impedes its interplay with apoptosis signal-regulating kinase 1 (Ask1) [65]. Moreover, Hsp27 can directly associate with the kinase domain of Ask1, leading to the inhibition of its activity and further blocking the MKK/Jnk cell death pathway induced by oxidative stress [64].

As shown on different cancer cell lines, including breast, lung and prostate cancer, Hsp27 modulates the activity of the Hippo pathway, which is a major tumor suppressor. Dysregulation of this pathway, also known as the Salvador–Warts–Hippo (SWH) pathway, occurs in numerous cancer types and is correlated with carcinogenesis initiation and tumor progression, as well as an increase in its invasiveness and metastatic potential [66]. In cancer cells, transcriptional coactivators, the Yes-associated protein (YAP) and its paralog TAZ (transcriptional coactivator with PDZ-binding motif) are translocated into the nucleus and activate the oncogenic cascades TGF-β/SMAD, WNT/β-Catenin and integrin-linked kinase (ILK) [58]. In contrast, in healthy cells, activated mammalian STE20-like protein kinases 1 and 2 (MST1/2) interact with large tumor suppressor kinases 1 and 2 (LATS1/2), which, through the phosphorylation of adaptor protein monopolar spindle-one binder kinase activator 1 (MOB1), phosphorylate YAP and TAZ, causing their retention in the cytoplasm [67]. Overexpressed in cancer cells, Hsp27 binds MST1, enhancing its ubiquitination and proteasomal degradation, which disrupts the phosphorylation cascade by reducing the activity of the downstream effectors LATS1 and MOB1 [59].

Through affecting and altering other signaling pathways, Hsp27 can promote the epithelial-to-mesenchymal transition (EMT) in cancer cells, which is basically an acquisition of mesenchymal features by malignant epithelial cells. This phenomenon implies changes in the cell morphology, proteomic profile and functional properties, and is a major driver of cancer cell dissemination from the primary tumor site, leading to the formation of metastases [68]. The revealed molecular mechanisms underlying the potential of overexpressed Hsp27 to enhance EMT to date involve the following: (i) the activation of the IL-6/STAT3/Twist pathway via inducing STAT3 phosphorylation, either through IL-6 or independently, as well as via the interaction with high-mobility group nucleosome-binding domain 5 (HMGN5) [69,70]; (ii) the promotion of β-catenin nuclear translocation and the upregulation of EMT activators through the EGF/β-catenin cascade [71]; (iii) the induction of the β-catenin/MMP3 pathway, significant in the course of the EMT due to the extracellular matrix-degrading activity of matrix metalloproteinases (MMPs); (iv) the upregulation of the TGF-β1/p38 MAPK pathway, inducing the synthesis of EMT-promoting growth factors and cytokines [72,73]; (v) the protection of Snail from proteasomal degradation and the subsequent facilitation of TGF-β1-mediated EMT [74,75]. Overexpressed Hsp27 has been reported to contribute to the maintenance of stem cell characteristics in esophageal cancer cells, dependent on the AKT/mTOR/HK2 pathway. Such features of cancer stem cells, such as the increased glycolysis rate and oxidative phosphorylation, have been attributed to the direct interaction of Hsp27 with Akt, leading to the upregulation of hexokinase 2 (HK2)—a major glycolytic enzyme mediating the Warburg effect [76]. Wei et al. [77] demonstrated that Hsp27 takes part in the maintenance of breast cancer stem cells in the mechanism of NF-κB activation and the regulation of IκBα degradation. 

In addition, the ability of Hsp27 to promote angiogenesis was described. Thuringer et al. [78] explored that, in breast cancer cells, Hsp27—through the interaction with Toll-like receptor 3 (TLR3)—can activate NF-κB, which, afterward, leads to an increase in vascular endothelial growth factor (VEGF) expression and induces the secretion of VEGF-activating VEGF receptor type 2. Thereby, it can be seen that Hsp27 indeed plays a multifaceted role in cancer, contributing to its progression in the numerous manners via supporting signal transduction through crucial pathways and enhancing cancer cell metastatic spread both by the EMT and the stimulation of angiogenesis, underlining its importance.

#### 3.1.3. Hsp27 Mediates Resistance to a Wide Range of Chemotherapeutics

**Figure 2:**
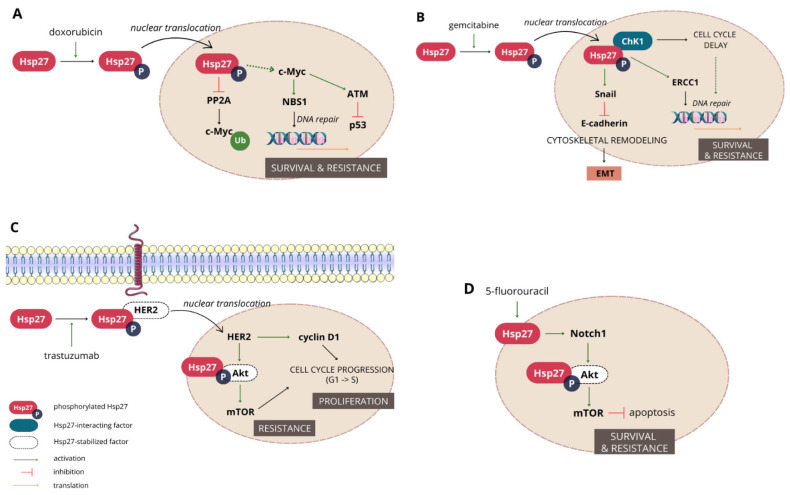
Representation of molecular mechanisms and cellular events involved in chemoresistance to certain chemotherapeutic agents mediated by Hsp27: doxorubicin (**A**), gemcitabine (**B**), trastuzumab (**C**) and 5-fluorouracil (**D**).

Hsp27 is acknowledged to have an important contribution to doxorubicin (DOX) resistance. Studies conducted in the last decades on different breast (MCF7, MDA-MB-231, MDA-MB-435), colon (CaCo2, HT-29) and prostate (LNCaP) cancer cell lines clearly link Hsp27 overexpression with decreased DOX-induced apoptosis [58]. A few mechanisms have been proposed to explain this phenomenon of Hsp27-mediated cancer cell protection. Based on the observation that Hsp27 suppresses p53 and p21, which is a cyclin-dependent kinase inhibitor, Ramani and Park [79] suggested that, as a result, the downregulation of downstream pathways dependent on p53 activity, such as NF-κB and STAT3, occurs, preventing cells from apoptosis. However, the status of p53 activity has previously been found to be related to the Hsp27 phosphorylation state rather than its total level, since only phosphorylated Hsp27 had an inhibitory effect on p53 and led to the evasion of apoptosis [80]. A recent study on MCF7 breast cancer cells gave consistent results and provided new insight. Bi et al. [81] have shown that the phosphorylation of Hsp27 preceded by exposure to DOX led to the upregulation of c-Myc in the nucleus, causing ataxia-telangiectasia mutated (ATM) kinase activation and which further negatively affected p53 (Figure 2).

Another agent in the resistance to which Hsp27 has been found implicated is trastuzumab, also known as herceptin, a monoclonal antibody targeting human epidermal growth factor receptor 2 (HER2), which is overexpressed in up to 30% of invasive breast cancer cases and also in some gastric cancer subtypes. This transmembrane tyrosine kinase receptor mediates the activation of intracellular signaling pathways associated with cell survival and proliferation, like PI3K/Akt or Ras/RAF/MEK/ERK. The HER2 status of a tumor highly determines the response to treatment, since it is associated with high aggressiveness; moreover, a high fraction of patients who initially responded to the trastuzumab therapy developed resistance to this agent during the first year of treatment [82]. Cell culture studies indicate that the inhibition of HER2 downregulation by this agent in chemoresistant cancers is related to the formation of the HER2–Hsp27 complex, protecting HER2 from trastuzumab and increasing its stability [83]. Later, it was found that the Hsp27 phosphorylation at the serine-15 residue is crucial in promoting HER2 and Hsp27 nuclear translocation, the induction of the survival Akt/MAPK/mTOR pathway and heat shock factor 1 (HSF1), the upstream transcriptional regulator of Hsp activation. Apart from Akt activation, nuclear HER2 also promotes cyclin D1 expression, the activity of which is associated with cell cycle progression and enhanced proliferation [84].

Transcriptomic and proteomic studies have experimentally connected the high expression of Hsp27 with resistance to gemcitabine, a primary drug used in the treatment of pancreatic cancer. The comparison of gemcitabine-resistant and sensitive pancreatic cells showed that, in the former cell line, Hsp27 is overexpressed, along with Snail and the DNA-binding zinc finger protein, while E-cadherin transcription is suppressed. These effects were reversed after Hsp27 downregulation via transfecting cells with Hsp27 small hairpin RNA [85]. Gemcitabine resistance was partially attributed to modifications in the cytoskeleton, such as a decrease in the E-cadherin level caused by Snail, which contributed to the EMT of cancer cells. Moreover, nucleotide excision repair after chemotherapy was observed due to the increased activity of excision repair cross-complementing protein-1 (ERCC-1) [75]. The downregulation of Hsp27 has also been proven to increase the cytotoxic properties of gemcitabine towards cancer cells in other studies [86,87], and its nuclear, but not cytoplasmic, localization has been confirmed to play a crucial role in resistance development [86]. Kang et al. [88] suggested that the ratio of phosphorylated to non-phosphorylated Hsp27 is the major determinant in predicting gemcitabine resistance. It was also shown that gemcitabine induces the activation of p38 MAPK, which, via the subsequent activation of respective kinases, phosphorylates Hsp27 while not influencing its total levels. Phosphorylated Hsp27 can further interact with checkpoint kinase 1 (Chk1), which, under stress conditions, delays cell cycle progression and thereby allows gemcitabine-induced DNA damage repair, eventually leading to resistance development [88,89]. On the other hand, somewhat contradictory results have been reported by Nakashima et al. [90], who showed that Hsp27 phosphorylated at the Ser15, 78 and 82 residues mediated gemcitabine-induced tumor growth suppression.

Accumulating evidence indicates the implication of Hsp27 in chemoresistance to 5-fluorouracil (5-FU)—a chemotherapeutic inhibiting thymidylate kinase and, subsequently, DNA replication, used in the treatment of various cancer types. Moreover, 5-FU is known to enhance apoptosis in cancer cells, mainly via downregulating the Akt/mTOR pathway, and inhibit their growth via p53-mediated cell cycle arrest in phase G1/S [91]. Several studies conducted on colon cancer cell lines, and later confirmed in animal models, indicate that cells in which Hsp27 is downregulated are more sensitive to 5-FU than control cells. This can be explained by the fact that Hsp27 suppression decreases both Notch1 expression and the Akt/mTOR phosphorylation level, influencing the downstream signaling pathway [92,93]. Apart from colon cancer cells, the overexpression of Hsp27 caused by exposure to 5-FU combined with carboplatin led to the decreased sensitivity of two different lines of hepatoma cells to 5-FU, while the addition of quercetin as an Hsp27 inhibitor, or downregulating its expression by specific siRNAs, sensitized both lines to 5-FU again [94]. 

### 3.2. Hsp40

#### 3.2.1. Hsp40s—A Diverse Co-Chaperone Family

The Hsp40 family, also referred to as DNAJ, stands out as the largest and most varied group of eukaryotic co-chaperones. There are three subfamilies of human Hsp40 (DNAJA, DNAJB and DNAJC), each containing several members, with a total of 49 distinct proteins. Additionally, each has several isoforms produced by alternative splicing [95]. The classification of particular Hsp40s into subclasses depends on their domain organizations [96]. Class I consists of an N-terminal J domain, a glycine/phenylalanine-rich region (G/F), a cysteine-repeat motif (Cys-repeat) and a largely uncharacterized C-terminal domain. Class II lacks the Cys-repeats, while class III contains neither G/F nor Cys-repeat regions in the polypeptide chain. The J domain can be positioned at any location within the protein, diverging from the exclusive N-terminal localization observed in classes I and II [97,98,99]. The third subclass is the most abundant and, at the same time, the least conserved group of human Hsp40s [100]. The J domain is the 70 amino acid sequence composed of four α-helices, predominantly functioning as an N-terminal end. The G/F region is believed to contribute to the stimulation of Hsp70 ATPase activity, but is not essential, since the selected type III Hsp40s can independently stimulate ATP hydrolysis without relying on this structure [101]. The Cys-repeat region induces protein folding in a zinc-dependent manner; as such, it is alternatively known as the zinc finger-like region [97,102,103]. Despite being largely undefined, the CTD is deemed essential for its dimerization and efficient co-chaperone role; furthermore, some of its specific regions can determine other protein functions [104].

Besides stimulating the ATPase activity of Hsp70 through its J domain, Hsp40s also transiently bind and deliver client peptide substrates to the ATP-bound form of Hsp70 chaperones, thereby assisting their function in folding, unfolding and translocating proteins. Interaction between the J domain and ATPase domain of Hsp70 induces ATP hydrolysis, and its ADP-bound form exhibits increased affinity for the protein substrate. Consequently, it forms a strong binding interaction with the unfolded protein, leading to the dissociation of Hsp40. Unfolded or misfolded protein aggregation or non-productive folding pathways are halted [105,106]. Hsp40 proteins also exhibit a particular and location-selective capability in presenting polypeptides to the Hsp70s [107]. Some representatives of this family function as co-chaperones for the Hsp90 chaperone machinery, forming multi-chaperone complexes [97].

#### 3.2.2. Less-Known Carcinogenesis Contributors

While there is a wealth of knowledge on the involvement of Hsp proteins in cancer development, the contribution of the Hsp40 family remains less explored. Nonetheless, it is confirmed that its specific members play a pivotal role in carcinogenesis, often functioning opposingly as both anticancer and procancer factors [105]. Certain Hsp40s exhibit pro-oncogenic activities in various human cancers, in several mechanisms, including the inhibition of p53-dependent apoptosis via destabilizing the programmed cell death protein (PDCD) [108], the promotion of the EMT [109], the promotion of cell cycle progression by inhibiting the ubiquitin degradation of some cell division cycle proteins or the hyperactivation of the pERK-IQ-domain GTPase-activating protein 1 (IQGAP1) signaling axis, thereby activating downstream oncogenic and metastatic pathways [106]. On the other hand, Hsp40 family member B4 has been reported to slow the cell cycle of lung cancer cells and participate in UV-related apoptosis, serving as caspase-3 substrate, while B6 partly reverses the mesenchymal phenotype in breast cancer cells and reduces malignant activity. This effect has been reported to rely on Wnt/β-catenin pathway inhibition via upregulating its protein inhibitor [110]. Liver cancer cell proliferation has been observed to be suppressed by the cytoplasmic Hsp40 family member C25. This factor is thereby suggested to have a role in hepatocellular carcinogenesis, since its overexpression induces the apoptosis of liver cancer cells, reducing the number of neoplastic colonies [111,112].

#### 3.2.3. Chemoresistance and Hsp40s

Due to Hsp40s’ involvement in carcinogenesis and cancer cell functionality, there have been significant attempts to assess its potential role in chemotherapy, as well as its impact on chemoresistance. In the case of hepatocellular carcinoma (HCC), which is still one of the most common solid tumors with a poor prognosis, studies were performed to investigate the correlations between the activity of standardly used chemotherapeutics and the expression of Hsp proteins using HepG2 and Hep3B cell lines. It has been shown that both 5-FU and carboplatin specifically induce the expression of Hsp27 and Hsp40 in the studied cells, indicating that the upregulation of these factors is a survival mechanism in response to treatment, which leads to the conclusion that the use of inhibitors targeting Hsp27/Hsp40 or the implementation of knockdown strategies for Hsp27/Hsp40, in conjunction with chemotherapeutic agents, could serve as a reasoned therapeutic approach for HCC [94]. This was supported by the finding that the pretreatment of malignant cells with quercetin, a biochemical inhibitor of Hsps (especially Hsp27 and Hsp40), reduced cancer cell survival, potentiating 5-FU and carboplatin-mediated hepatoma cell death. Also, quercetin causes considerable antiproliferative effects in neuroblastoma cells and sensitizes them to doxorubicin. This can be partly attributed to the inhibition of Hsp expression via the depletion of heat shock factor 1 (HSF1) cellular stores [113].

The expression of Hsp40 family member B8 (DNAJB8) has been observed to increase in cancer stem-like cells, and it has been discovered that this representative of Hsp40s plays a key role in the progression of human renal cell carcinoma and resistance to chemotherapy [114]. In vivo studies confirmed that cells with the DNAJB8 knockout showed a reduced tumor initiation ability compared to wild-type cells and increased sensitivity to docetaxel, while they have not been sensitive to other stress factors such as low pH, low glucose or heat shock [114]. The small amount of available literature regarding the Hsp40 contribution to cancer and chemoresistance in comparison to other Hsps, such as Hsp27 or 70, points out that this topic is yet to be deeper explored to provide more detailed and comprehensive information. 

### 3.3. Hsp60

#### 3.3.1. Hsp60—Mitochondrial Chaperonin

Hsp60, also known as the 60 kDa chaperonin (Cpn60), belongs to stress-induced Hsps and, due to its main site of localization, is referred to as a mitochondrial matrix molecular chaperone [115]. Similarly to other Hsps, its typical function includes the proper folding of the imported pre-proteins to reach their native conformation and the restoration of the misfolded protein structure due to stress, also with the assistance of mitochondria-residing Hsp10 as its co-chaperone [116]. Hsp60 can exist in the following three forms: monomers, single rings and double rings [117], with a single heptameric ring as its main form [118]. According to X-ray crystallography studies, ATP converts into a tetradecameric double-ring structure in the presence of ATP, which can interact with the Hsp10 heptamer, forming a football-like complex [119]. There, Hsp10 forms a lid enclosing the client polypeptide inside the central cavity, where it is isolated and protected from the external environment to be properly folded [120]; it has also been shown to control the interactions between the Hsp60 monomers as well as the hydrolysis of ATP [121]. Moreover, the Hsp60/Hsp10 complex, through maintaining the steady state of mitochondrial proteins, manages the functionality of the respiratory chain. Many mitochondrial matrix proteins have been found to interact with the Hsp60/Hsp10 quality control system to ensure cell survival [122].

Although the majority of the Hsp60 pool, due to its essential role in maintaining cellular proteostasis, resides in the mitochondria, it can also be found in smaller amounts in the cytoplasm, where it resides along with Hsp10, the surface of the cell membrane, exosomes released from the cells and the extracellular matrix. This distribution concerns both healthy and tumor cells, and is suspected to be associated with transport through cell membranes and signal transduction processes [123]. In addition to the canonical processes regulated by Hsps, other than the broadly defined protective functions on protein conformation [124], i.e., the regulation of apoptosis and the proliferation of cells, Hsp60 also participates in immune responses and inflammatory reactions because it serves as a ligand for Toll-like receptors and an antigen for lymphocytes T and B [125].

#### 3.3.2. Hsp60 as a Player in Cancer Progression

Hsp60 is involved in both the extracellular interactions and intracellular signaling pathways of tumor cells. The overexpression of Hsp60 has been noticed in a variety of cancers, including breast, colorectal, prostate, ovarian, pancreatic and non-small-cell lung cancer, or hepatocellular carcinoma. High levels of this actively secreted molecule have been connected to worse patient prognosis [126]. There is a discrepancy between the known fact that Hsps are mostly proteins promoting cell survival and the observations that Hsp60 can exhibit the opposite activity [122]. Mitochondrial and cytosolic Hsp60 were shown to be implicated in both pro-survival and pro-apoptotic pathways, and it has been reported to depend on differential interaction with caspase-3. Exposure to certain apoptotic stimuli can cause either the rapid release of Hsp60 from the mitochondria to the cytosol, where it is supposed to participate in caspase-3 activation, or its accumulation in the cytosol. The experimental data suggest that the former scenario with apparent Hsp60 outflow is pro-apoptotic and leads to fast cell death, whereas, in apoptotic systems lacking significant mitochondrial Hsp60 release but with its accumulation in the cytosol, the progress of apoptosis is slower, indicating its pro-survival role [127]. According to the study on HeLa cells, this promotion of cell survival has been linked to Hsp60 involvement in the NF-kB pathway [128].

The anti-apoptotic role of Hsp60 is also manifested by its ability to form complexes with Bax and Bak, and subsequently block their activity, which is related to cancer cell survival [129,130]. Moreover, it has been recently discovered on colon and prostate cancer cells that Hsp60 can upregulate the expression of anti-apoptotic interleukin-8, either directly or via TGFβ, promoting the viability of tumor cells [131]. The interactions of Hsp60 with several cancer-related proteins and apoptosis regulators, such as survivin, p21 and p53, have also been described [132]. Mitochondrial survivin, highly expressed in cancer, by inhibiting pro-caspase activation, prevents the apoptosis of tumor cells and has been found to be stabilized and prevented from degradation by Hsp60 [133]. Other pro-carcinogenic functions of Hsp60 include inhibiting mitochondrial permeability, blocking mitochondrial ROS release and ROS-mediated cell death [126]. An additional mechanism discovered in neuroblastoma cells was the direct inhibition of tumor-suppressive clusterin activity [134]. Noteworthy, Hsp60 seems to be associated with the acquisition of the mesenchymal phenotype of cancer cells. As reported by Tsai et al. [135], the interaction of Hsp60 with β-catenin results in its activation, which has a metastasis-inducing effect. Another hallmark of cancer with the involvement of Hsp60 is the sustained proliferation, mediated by this protein mainly by interacting with the following two signaling pathways: the mTOR and MAPK cascades [136].

#### 3.3.3. Hsp60 and Drug Resistance

Knowing that Hsp60 exhibits anti-apoptotic activity in a number of cancer types, it has been suggested that it can have a role in drug resistance development. Indeed, the overexpression of Hsp60 has been found to be associated with resistance of ovarian and bladder cancer cells to the platinum compounds cisplatin and oxaliplatin in comparison to their parental cells [137]. Additionally, in these cancers, the degree of resistance to platinum analogs was found to be correlated with Hsp60 mRNA expression levels [137]. Experiments on head and neck cancer cell lines gave similar findings [138]. However, the exact molecular basis of Hsp60 involvement in resistance to platinum compounds remains unresolved. It is proposed that, like other Hsps, Hsp60 can play a role in cellular response regulation and the detoxification of the drug, along with GSH-related enzymes and ribosomal proteins [139]. Earlier studies report that the overexpression of both Hsp60 and Hsp10 in a HeLa-derived cell line resulted in higher resistance to cisplatin [140]. 

Another group of chemotherapeutics for which the resistance has been linked to Hsp60 overexpression are taxanes, as shown in the example of tamoxifen and breast cancer cells [141]. Altogether, these discoveries are of importance on account of the novel therapeutic approaches aiming to restore cancer sensitivity to chemotherapeutics; for example, the combined treatment of cisplatin or docetaxel and the Hsp60 antibody led to a synergistic therapeutic effect on ovarian cancer cells of an epithelial origin [142]. Also, as demonstrated in vitro, Hsp60 contributes to the 5-fluorouracil resistance of colorectal cancer cells, and the inhibition of its expression results in the sensitization of the cells to the drug [143]. In the light of the provided data, the role of Hsp60 in cancer cell apoptosis inhibition and, subsequently, chemotherapy resistance development is undeniable. Thereby, the potency of Hsp60/Hsp10 targeting shall be further investigated as a therapeutic approach.

### 3.4. Hsp70

#### 3.4.1. Hsp70 Protein Family

Hsp70, with its stress- and non-stress-inducible members, is the best-characterized family within the group of Hsp proteins, which, over the years, has gained attention in cancer biology due to its vital role in maintaining cellular homeostasis, its impact on apoptosis and for protecting cells against various stressors [144]. The 70 kDa chaperone family members are distributed throughout nearly all subcellular compartments of nucleated cells and present the structure most characteristic for ATP-dependent Hsps, since two major functional domains, NBD and SBD, can be distinguished in their architecture [62,144]. NBD is approximately 44 kDa in size and functions as an ATP-binding domain, while SBD can be further divided into two subdomains. The 25 kDa N-terminal subdomain of the β-sandwich structure forms the substrate-binding pocket and interacts with extended polypeptides, acting as a receptor for client proteins. The second, a 10 kDa C-terminal subdomain, comprises five α-helical structures and functions as a flexible lid that can cover the substrate-binding pocket, regulating access to client proteins and influencing their interactions. The NBD and SBD domains of Hsp70 are interconnected by a flexible linker with a highly conserved leucine-rich motif (LRR) that plays a critical role in facilitating the recruitment of Hsp40 as a co-chaperone [144].

The co-chaperones associated with Hsp70 can be categorized into three types. J-domain co-chaperones are represented by Hsp40, which binds to the ABD and stimulates its ATPase activity. Nucleotide exchange factors, such as Bag-1, Hsp110 or HspBP1, facilitate the release of ADP from Hsp70, completing the ATPase cycle of Hsp70 [145]. Tetratricopeptide repeat (TPR) domain chaperones like Hop and CHIP bind to the C-terminal sequences and are crucial for assembling Hsp70 and Hsp90 complexes [5]. Hsp70 is crucial in facilitating protein folding, disaggregation, the transportation into destined subcompartments, the assembly of multiprotein complexes and degradation processes by serving as a physical platform for binding client proteins and other chaperones and co-chaperones. The ultimate fate of the client protein is determined by the specific set of interactions that take place with Hsp70 in a particular cellular context [146].

#### 3.4.2. Hsp70 in Cancer

**Figure 3:**
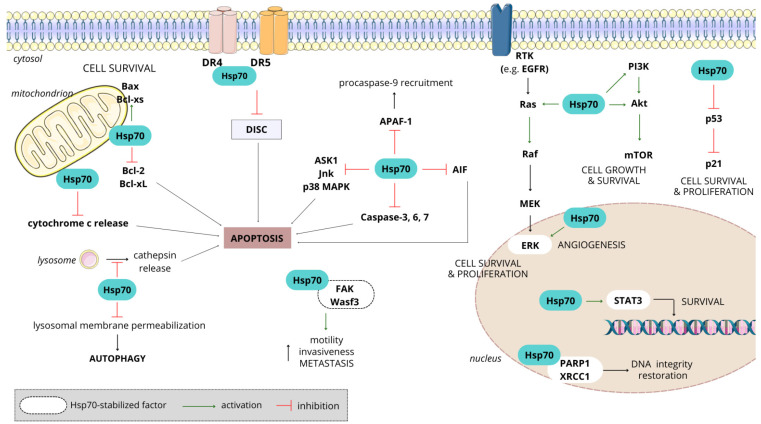
Signaling pathways and molecular events associated with Hsp70’s role in cancer progression. Hsp70 is involved in multiple means of apoptosis evasion, through inhibiting a wide array of pro-apoptotic factors and promoting anti-apoptotic cascades, leading to cancer cell growth, survival and proliferation, as well as increasing their motility and invasiveness. DR—death receptor; RTK—receptor tyrosine kinase; EGFR—epidermal growth factor receptor; PI3K—phosphoinositide-3-kinase; mTOR—mammalian target of rapamycin; MEK—mitogen-activated protein kinase; ERK—extracellular signal-regulated kinase; STAT3—signal transducer and activator of transcription 3; PARP1—poly [ADP-ribose] polymerase 1; XRCC1—X-ray repair cross-complementing protein 1; APAF1—apoptotic protease activating factor 1; AIF—apoptosis-inducing factor; ASK1—apoptosis signal-regulating kinase 1; Jnk—c-Jun N-terminal kinase; p38 MAPK—p38 mitogen-activated protein kinase; DISC—death-inducing signaling complex; FAK—focal adhesion kinase; Wasf3—Wiskott–Aldrich syndrome protein family member 3; Bcl-2—B-cell lymphoma 2; Bax—Bcl-2 associated X protein; Bcl-xL—B-cell lymphoma-extra-large.

Hsp70 is commonly observed to be upregulated in various types of tumors. Compared to other stress proteins within the Hsp family, the production of Hsp70 is faster, accumulating to higher levels in tumor cells when exposed to environmental stress [147]. The heightened expression of Hsp70 in cancer cells could potentially be accountable for tumorigenesis and progression, and may confer resistance to chemotherapy [148]. Elevated cytosolic levels of Hsp70 were proven to protect cancer cells against apoptotic death, enhance tumor cell proliferation and migration, facilitate resistance to therapy and contribute to an aggressive tumor phenotype [147]. Hsp70 overexpression has emerged as a poor prognosis marker, showing strong correlations with the clinical stage and overall survival in a diverse array of human cancers, including lung, breast, colon, liver, prostate, esophagus and cervix [144]. In cancer, Hsp70s play an important role in the following processes: (i) resisting cell death and sustaining proliferative signaling, (ii) evading growth suppression, (iii) escaping immune destruction, (iv) providing replicative immortality, (v) supporting tumor-promoting inflammation, (vi) activating local invasion and promoting angiogenesis and metastasis and (vii) deregulating the energetic metabolism at the cellular level [145] (Figure 3).

Interestingly, in vitro and in vivo studies show that Hsp70 possesses a dual function. On the one hand, intracellular Hsp70 exerts a cell-protective role by inhibiting apoptotic pathways and lysosomal cell death, and extracellular forms of Hsp70 have been associated with promoting tumorigenesis. It affects processes such as the release of cytochrome c, the activation of caspases, the accumulation of misfolded proteins, the generation of reactive oxygen species, as well as DNA fragmentation, and the inhibition or knocking down of Hsp70 enhances the sensitivity of cells to apoptosis [148]. The immunogenic properties of Hsp70 stem from its capacity to bind tumor-derived antigenic peptides [145]. Tumor cells expressing Hsp70 on their cell membranes actively release exosomes that also bear Hsp70 on their surfaces, triggering the activation of NK cells [149]. Functioning as a DAMP (damage-associated molecular pattern), Hsp70 released from dying cancer cells possesses significant immunogenic potential, capable of generating robust anti-tumor T-cell responses [145]. However, the prolonged exposure of immune cells to free Hsp70 after radiotherapy has been shown to cause immune tolerance and facilitate tumor growth [150]. These observations align with studies indicating that, even a low dose, the Hsp70–peptide complex is enough to stimulate anti-tumor immunity [145]. Applying this to naturally released Hsp70 from tumor cells, it is conceivable that the initial Hsp70 release has a tumor-suppressing effect, while an excessive Hsp70 load contributes to tumor progression. On the other hand, intracellular Hsp70 can induce apoptosis, and membrane-associated/extracellular forms of Hsp70 have the potential to stimulate adaptive immune responses against tumor cells [151].

#### 3.4.3. Significance of Hsp70 in Chemotherapy Resistance

The cell-preserving mechanisms induced by Hsp70 indicate its role in resistance to chemotherapy. For instance, the overexpression of Hsp70 in the imatinib-sensitive K562 cell line resulted in the development of resistance to this agent, observed as a significant decrease in cell death [152,153]. Elevated levels of Hsp70 seem to confer a protective effect against the cytotoxic activity of imatinib, thus promoting cell survival in the presence of the drug. Findings proved that imatinib- and nilotinib-resistant K562-r present increased Hsp70 expression in comparison to imatinib-sensitive parental K562 cells (K562-s), and that the inhibition of this expression leads to and is sufficient for a 34% reduction in cell viability in the presence of imatinib. The resistance of K562-r cells persisted despite a full block of the kinase Bcr-Abl activity, suggesting that the resistance mechanism is independent of its activity. Moreover, it was proven that the initially low Hsp70 expression in imatinib-susceptible patients drastically increased following the development of imatinib resistance and blast crisis [152]. In addition, Hsp70 has been determined to be involved in the resistance to ibrutinib, an inhibitor of Bruton’s tyrosine kinase (Btk) in chronic lymphocytic leukemia (CLL), as shown in vitro on leukemic cells obtained from untreated patients and those in which the ibrutinib treatment failed [154]. 

Based on the available data, Hsp70 mediates resistance to several widely used classical antitumor agents. Direct evidence for gemcitabine resistance development in pancreatic ductal adenocarcinoma was provided by Wang et al. [155]. The authors demonstrated that protein arginine methyltransferase 1 (PRMT1) serves as a substrate for Hsp70, and its methylation performed by this enzyme promotes the ability of Hsp70 to bind and stabilize Bcl2 under stress conditions, including chemotherapy. This results in the Bcl2-mediated apoptosis evasion of cancer cells and leads to the further development of a resistant phenotype [156]. Resistance to gemcitabine and topotecan mediated by overexpressed Hsp70 was also reported for fibrosarcoma [157].

The synthesis of Hsp70 was observed to be upregulated in HT-29-resistant colon cancer cell lines after exposure to 5-FU. The collected data indicate that the accumulation of this chaperone promotes cytoprotective effects against apoptosis induced by 5-FU, with great probability in influencing the mitochondrial pathway of apoptosis and inhibiting caspase-dependent events, such as cytosolic phospholipase A2 activation and nuclear morphology activation [158]. Regarding colon cancer, another interesting result was obtained by Feng et al. [159], who demonstrated that Hsp70 can activate Toll-like receptor 2 (TLR2) and further influence the immune microenvironment of tumor cells, inducing an immunosuppressive response counteracting CD8+ T-cell function. Such remodeling translates into the loss of sensitivity to combined treatment with oxaliplatin and 5-FU, but the exact mechanism remains unclear. To investigate the role of Hsp70 expression in developing resistance to paclitaxel, siRNA knockdown in chronic myeloid leukemia cell lines was performed, leading to a reduction of cytoplasmic Hsp70 levels by 50%. The impact of Hsp70 on cell survival was measured by the apoptosis rate in cells cultured in the presence or absence of paclitaxel. In comparison to the control cells, the Hsp70-transfected cells did not exhibit any significant changes in the spontaneous rate of apoptosis, whereas these cells in which Hsp70 was downregulated showed an enhanced apoptotic rate in response to paclitaxel [160]. The collective data strongly suggest that the upregulation of Hsp70 plays a role in developing paclitaxel resistance. Another interesting relationship was observed between methotrexate (MTX) resistance and Hsp70, since it was observed that the phosphorylation status of Hsc70—a member of the Hsp70 family—regulates the transport of this drug to the cells via the reduced folate carrier system and contributes to MTX resistance in murine leukemia L1210 cells [161].

Research aiming to investigate the impact of inductive therapy used in breast cancer on Hsp70 expression and to explore its potential influence on drug resistance found a significant increase in nuclear expression coupled with a decrease in the cytoplasmic expression of the protein following chemotherapy. Furthermore, tumors from patients who developed drug resistance exhibited a statistically significant increase in the expression of Hsp70 within the nuclear compartment. Moreover, a substantial nuclear presence of Hsp70 in tumor cells (>10%) was significantly associated with resistance to chemotherapy that was performed with 5-FU, DOX, MTX, cyclophosphamide and epirubicin in various doses and combinations [162]. The resistance of ovarian cancer cells to cisplatin therapy is a well-known concern, with the established role of Hsp70 as a factor preventing Bax translocation to the mitochondria. Since Bax is indispensable for mitochondrial proteins, including cytochrome c, release and apoptosis induced by cisplatin to take place, its binding by Hsp70 results in cancer cell survival and chemoresistance development [163]. In addition, as shown on gastric cancer cells (HGC-27), Hsp70 facilitates the activation of the MAPK pathway as a response to cisplatin, thereby protecting them from cisplatin-induced apoptosis [164]. Recently, the resistance of osteosarcoma cells to cisplatin mediated by the Hsp70-modulated Jnk/Jun downstream signaling pathway has been described [165]. The contribution of Hsp70 to cisplatin resistance was also demonstrated in animal models [166].

Considering the issue of chemoresistance, the role of exosomes—a type of extracellular vesicle (EV) serving as information messengers—should not be neglected. Indeed, Hsps are often found in considerable amounts both inside and at the surface of the vesicular membranes of exosomes secreted by cancer cells [167]. Hu et al. [168] demonstrated that EVs containing Hsp70, which were secreted by DOX-resistant MCF-7 cells, conferred the resistant phenotype to previously sensitive cells. The authors revealed that, in this case, Hsp70 was delivered to the mitochondria of the sensitive recipient cells, impairing the mitochondrial respiratory chain and inducing metabolic reprogramming, steering it into the glycolysis pathway. With such extensive and detailed knowledge concerning Hsp70 involvement in cancer progression and drug resistance, gathered throughout decades of extensive research, this protein arises as a major anticancer therapy target whose inhibition would prove beneficial at multiple levels.

### 3.5. Hsp90

#### 3.5.1. Hsp90—Another ATP-Dependent Chaperone

Besides participation in processes such as folding, stability maintenance, activation and the proteolytic turnover of a vast array of proteins, which are typical for Hsps, Hsp90 also takes part in their post-translational modifications, such as acetylation, phosphorylation or S-nitrosylation [169]. In cancers, Hsp90 upregulation allows cells to survive and maintain protein homeostasis in stress conditions like hypoxia or a lack of nutrition, which prevail in the tumor microenvironment [170]. Apart from Hsp90, which occurs in two isoforms (cytosolic α and β), two more members of this family are distinguished—tumor necrosis receptor-associated protein 1 (TRAP-1), located in the mitochondria, and 94kDa glucose-regulated protein (Grp94), localized in the endoplasmic reticulum [169]. Hsp90 is a homodimer consisting of monomers comprising three main functional domains—NBD, SBD and a middle domain connected to the N terminus by a charged linker region, which increases the protein’s flexibility and dynamics [1,169]. Hsp90 cycles between the closed and open states: the binding of ATP to NBD takes place in an open conformation, inducing its complex conformational rearrangement. The hydrolysis of ATP and the following replacement with ADP results in the release of stabilized and activated client proteins and the return of Hsp90 to its original conformation [171].

#### 3.5.2. Roles of Hsp90 in Carcinogenesis

Cancer cells release Hsp90 into the microenvironment, where it can interact with several co-chaperones in order to form complexes able to perform specific functions, which range from the proper folding and activation of extracellular clients to stimulating the receptors present on the cell surface to induce signal transduction pathways [172]. Stabilizing the structures of protein kinases, receptors and transcription factors mediates cell growth and proliferation, leading to tumor progression [173]. Hence, Hsp90 was found overexpressed in many hematological and solid cancer types, such as leukemias, breast, lung, prostate and ovarian cancers, among others. On the protein level, it plays the role of a biochemical buffer for many genetic mutations present in tumor cells, allowing for the achievement or maintenance of the functions of mutated proteins while permitting cancer cells to tolerate the impaired signaling mediated by these oncogenic factors, and thereby to escape apoptosis [174]. A panel of Hsp90 clients includes several proteins involved in extracellular matrix remodeling, like metalloproteinases (MMP2 and MMP9), the pro-form of tissue plasminogen activator (tPA), the lysyl oxidase-like protein 2 (LOXL2) and fibronectin. This suggests that extracellular Hsp90 regulates the matrix deposition and stiffness, thereby influencing cancer aggressiveness [175]. Other oncogenic proteins which can be listed among the Hsp90 substrates are p53, Bcr-Abl, Akt, Her-2, Cdk4, Cdk6, Raf kinase and Src in tumor cells [24]. Concerning surface molecules, Hsp90 binds with Toll-like receptor 4 (TLR4) and communicates by the proto-oncogene tyrosine-protein kinase (SRC) to focal adhesion kinase (FAK), which is critical for cell motility. The connection between Hsp90 and TLR4 can lead to the transactivation of the epithelial growth factor receptor (EGFR), further contributing to malignancy [172]. Interaction between Hsp90 and the extracellular domain of HER2/ErbB2 through the activation of the Akt, SRC and ERK pathways can lead to breast cancer cell invasion [176].

The role of Hsp90 in different cancers has been established by a number of studies. Accordingly, CRC Hsp90 was claimed to be responsible for promoting the EMT, migration and invasiveness of cells, as well as enhancing STAT3-mediated VEGF transcription to promote angiogenesis. In ovarian cancer, the interaction of Hsp90 with lamin-A allows DNA damage repair and is connected with the chemoresistance of cancer cells [20].

#### 3.5.3. Underexplored Issue of Hsp90 Contribution to Chemoresistance

Studies concentrating on the elucidation of the Hsp90’s role in chemoresistance are limited. Yet, the ability of Hsp90 to stabilize a transcriptional repressor Snail in response to DNA damage suggests that it can be a mechanism in which Hsp90 contributes to the chemotherapy resistance of cancer. The activation of Snail and the downstream complex signaling network is known to induce several cellular events, including metabolic reprogramming, the EMT and tumor metastasis, which overall promote multidrug resistance [177,178]. Also, the protection and further activation of Akt, a known substrate of Hsp90, in a response to chemotherapy is well established to confer the acquired drug resistance of cancer cells [179]; similarly, the stabilization of the Raf kinase may result in the enhanced activation of the Raf/MEK/ERK pathway, which can govern drug resistance [180].

To date, the best studied association of Hsp90 with chemoresistance was established on multidrug-resistant ovarian cancer cells. Yin et al. [181] demonstrated that Hsp90 is involved in the positive regulation of P-gp, breast cancer resistance protein (BCRP), survivin and Bcl-2, which are closely connected with drug resistance, as well as in β-catenin accumulation and Akt/GSK3β signaling activation, altogether leading to the resistance to paclitaxel and cisplatin. Using the epidermoid tumor cells model, Kumar et al. [182] have shown in the example of a geldanamycin derivative that increased activities of P-gp and Hsp90 mediate the chemotherapeutic drug adaptation of cancer. The role of Hsp90 appears to rely on facilitating cholesterol redistribution, which is mandatory for enhanced drug efflux activity, but, according to the authors, is not directly involved in P-gp activation. The finding that the inhibition of Hsp90 restores the sensitivity of pancreatic cancer cells to 5-FU and gemcitabine suggests that it is highly implicated in chemoresistance to these agents [183]. Hsp90 was also assessed as connected to the melphalan resistance of multiple myeloma, with the underlying mechanism indicated as Src kinase, an Hsp90 client, activation and its downstream signaling effects, including ERK, Akt and NF-κB induction [184]. On multidrug-resistant cancer cells, Hsp90 was shown to induce the expression of ABC transporters, such as P-gp, and the anti-apoptotic proteins survivin and Bcl-2, contributing to a lowered sensitivity to chemotherapeutics and apoptosis, whereas, in the case of Hsp90 inhibition, the observed effects were the opposite [181]. Based on the literature review, it can be seen that Hsp90 plays a key role in maintaining homeostasis and supporting the function of many proteins involved in cell growth, division and survival. This has made it a significant target for therapeutic intervention. For example, Hsp90 inhibitors have the potential to disrupt multiple signaling pathways in cancer cells, making them a promising area of research for cancer therapy.

### 3.6. Hsp110

#### 3.6.1. Hsp110—Not Only a Co-Chaperone for Hsp70

Proteins from the Hsp110 family, sometimes called Hsp105, are chaperones with anti-aggregation properties which contribute to maintaining protein homeostasis in synergistic action with proteins from the Hsp70 family [185]. Apart from Hsp110, the best-studied protein from which the entire family is named, cytoplasmic chaperones such as Apg-1 and Apg-2, and the endoplasmic reticulum chaperone Grp170 are classified as Hsp110 family members [186]. Hsp110 is structurally and functionally related to Hsp70, and shares a conserved nucleotide-binding domain, but has an extended C-terminal domain and an acidic region inserted between the elongated C terminus and the β-sheet subdomain [187]. Also, the loop connecting the β-sandwich and α-helical lid domains in Hsp110 is formed by a longer polypeptide chain than in Hsp70, and it is suspected to be of functional importance, since Hsp110 is not able to mediate protein folding autonomously [188]. Hsp110 acts as a holdase, binding and protecting unstable proteins from aggregation, and moreover is also a nucleotide exchange factor (NEF) regulating ATPase and the conformational Hsp70 cycle. By interacting with its NBD, Hsp110 promotes the ADP to ATP exchange, dissociating an Hsp70–client protein complex and protein release. Hsp110 and Hsp70 form stable heterodimers, connected through their NBDs, able to efficiently disaggregate even large, stable aggregates [189,190].

Another important fact is that Hsp110 can influence the Wnt/β-catenin pathway, which relies on signals dependent on Wnt family proteins through the canonical pathway (β-catenin-dependent). Hsp110 is expected to be needed for the Wnt protein-induced transcription of the target gene [48]. Hsp110 also supports the function of transcription factors, including the STAT3 factor [191]. It is also secreted to the extracellular environment, where it exhibits immune properties. It has been shown that, in inflammatory bowel diseases, the amount of Hsp110 in the gastrointestinal lumen increases [192].

#### 3.6.2. Dual Role of Hsp110 in Cancer

Some studies suggest that Hsp110 may have both protective and potentially harmful effects in the context of cancer [193]. On the one hand, heat shock proteins such as Hsp110 may help cancer cells survive and adapt to stressful conditions, such as those encountered during rapid tumor growth. They may assist with protein folding, prevent protein aggregation and contribute to the overall stress response of cancer cells [194]. On the other hand, there is evidence that Hsp110 may contribute to tumor progression by promoting cell survival, inhibiting apoptosis and supporting the development of more aggressive tumor phenotypes [195]. The overexpression of Hsp110 has been observed in various types of cancer. It is important to note that the specific role of Hsp110 in cancer treatment may vary depending on the tumor type and cellular context. The relationship between heat shock proteins and cancer is complex and may involve many factors [186,193].

Hsp110 is highly expressed in many cancer tissues, including melanoma, prolactinoma, pituitary adenoma, breast cancer, pancreatic cancer, colorectal cancer (CRC) and many others [196]. Considering that Hsp110 helps maintain the homeostasis of the body’s proteins, and its role in proliferative pathways, its association with aggressive cancers seems obvious. Studies in vitro and in vivo in CRC patients and colorectal cancer cells have shown that Hsp110 co-localizes with DNA damage and, in response to chemotherapy, is translocated to the nucleus, where it interacts with the non-homologous end-joining (NHEJ) repair machinery elements [197]. In addition, Hsp110 induces CRC growth by activating the STAT3 factor. These proteins are also most likely involved in the development of colon cancer by inducing the proliferation of cancer cells. This effect is also associated with the activation of the STAT3 factor. In the indicated condition, there is an increase in STAT3 phosphorylation through direct binding to Hsp110, nuclear translocation and the activation of other transcription factors. At the same time, the proliferative effect of Hsp110 blocks STAT3 inhibition. STAT3 regulates several pathways critical for cancer metastasis, including cell proliferation, invasion and angiogenesis; thus, Hsp110 may influence apoptosis and cancer development. RNA interference targeting the Hsp110 gene induced apoptosis in cancer cells, further confirming the role of this protein in inhibiting cancer cell death [196,198].

The role of Hsp110 family proteins in the Wnt/β-catenin signaling pathway has already been mentioned. It is also known that genes encoding Wnt proteins also contribute to cancer development. The conducted research has shown that the development and growth of breast cancer are directly dependent on Wnt-1 [199]. The inhibition of Wnt-2 activity induces apoptosis in non-small-cell lung cancer and melanoma. In turn, the overexpression of Wnt-5a increases the invasiveness and mobility of metastatic cancer cells. Moreover, Wnt-5a expression is strongly correlated with the stage of cancer progression. β-catenin also plays the role of an oncogene in many types of cancer. Mutations of its gene in the N-terminus lead to increased protein stability, protecting it against degradation. These mutations have been detected in cancers of the intestine, ovary, endometrium, pancreas, prostate, stomach, head and neck [200].

#### 3.6.3. Hsp110 as a Driver of Drug Resistance

Compared to other members of the Hsp family, the biological significance of Hsp110 is less understood. However, Hsp110 is abundant in CRC tumor cells and contributes to drug resistance [195,201]. The previous subsection mentioned the role of Hsp110 in the Wnt/β-catenin signaling pathway. This corresponds to the fact that cells characterized by the increased expression of the gene encoding Wnt-1 are resistant to apoptosis induced by chemotherapeutic drugs. Wnt-1 is supposed to inhibit the release of cytochrome c and also block the proteolytic activity of caspase 9, the action of which is induced by chemotherapy drugs. This is related to an increase in the transcription of oncogenes that depend on β-catenin [202,203].

In turn, Yamane et al. [204] showed that DOX, the DNA-damaging agent, induces the accumulation of Hsp110 in the nucleus through the reduced expression of CRM1, which is an export receptor for leucine-rich nuclear export signals. The knockdown of Hsp110 using small interfering RNA (siRNA) increases the sensitivity of cells to DOX, suggesting that this family of proteins may be responsible for inhibiting apoptosis in the nucleus [186]. The same is true for another drug—oxaliplatin. In response to treatment with this agent, Hsp110 also accumulates in the nucleus, and Hsp110 knockdown increases oxaliplatin-induced apoptosis [197]. However, studies show that the knockdown of Hsp110 will not always be effective in supporting chemotherapy. This effect may also reduce the sensitivity to the drugs used, as in the case of the agent targeting microtubules—paclitaxel [186]. Thereby, more research focused on elucidating the role of Hsp110 in resistance to specific anticancer drugs and the underlying molecular bases is necessary to fill the knowledge gaps. In particular, it would be essential for further translational studies aiming at the development of clinical applications.

Interestingly, Hsp110 is the only Hsp for which mutations were found in cancer, and such mutations in the genes encoding proteins from the Hsp110 family may also influence treatment resistance. Patients with CRC and rectal cancer who had an Hsp110-inactivating mutation, Hsp110∆E9, presented a better response to chemotherapy [185]. The use of information about the Hsp110∆E9 mutation may therefore contribute to the introduction of more personalized treatments for patients. Table 1 below, summarizes heat shock proteins associated with resistance to specific chemotherapeutic agents, detailing the cell lines used in the studies that confirmed these associations.

## 4. Therapeutic Strategies

Owing to their multifaceted roles in cancer progression, Hsps have naturally come under consideration as targets for innovative cancer therapies, which also include attempts to combat chemotherapy resistance. Therapeutic approaches focusing on Hsps can be based either on their direct inhibition or the translation of Hsps’ capability to trigger immune responses into immunotherapeutic factors in various cancer types, including developing anticancer vaccines [205].

### 4.1. Inhibitors of Heat Shock Proteins in the Fight against Cancer

#### 4.1.1. Hsp27 in Therapeutic Strategies

Since Hsp27 is a key player in cancer development due to its ability to protect tumor cells from apoptosis, as outlined in Section 3.1.2, it has become an important and promising therapeutic target. In several cancers, such as head and neck squamous cell cancer, and glioma, breast and lung cancers, positive effects after Hsp27 inhibition have been evidenced [206]. To reach the aim of reducing Hsp27 expression or inhibiting its activity, three approaches have been proposed.

In the first place, the idea of small-molecule inhibitors has been developed; however, the highly disordered structure of Hsp27 is a limiting factor for the rational design of structure-guided drugs [207]. A virostatic nucleoside brivudine (BVDU) and its derivatives exhibit high clinical potential among direct inhibitors. Hsp27 inhibition by BVDU relies on the direct binding between the drug and protein’s phenylalanine residues, which impairs the interaction of Hsp27 with Akt1, procaspase-3 and cytochrome C, and, as a result, reduces apoptosis [208]. Small molecules, synthetic or plant-derived, have also been employed to alter the dimerization of Hsp27 through the induction of cross-linking, when the covalent bond is formed between the cysteine and thiol groups. The phosphorylation of Hsp27, as a crucial step of its activation, can be targeted as well, e.g., by ivermectin, which binds to the “phosphorylation pocket” between NTDs, surrounded by serine residues, and blocks the interactions with client oncoproteins and pro-survival signaling pathways [207]. Quercetin, a plant-derived flavonoid, has been shown to downregulate Hsp27 expression and inhibit its function in oral squamous cell carcinoma cells [209] and breast cancer [210]. The molecular basis is supposed to rely on the inhibition of casein kinase 2 (CK2), the knockdown of which promotes Hsp27 proteasomal degradation [211]. In addition, quercetin can impair the phosphorylation of Hsp27 in cancer stem cells [209]. Although it has been proven by numerous studies that quercetin exhibits anticancer activity, as shown on cell lines representing various cancer types, as well as different cancer stem cells, its clinical utility is still arguable [212].

Secondly, antisense oligonucleotides targeting the mRNA of Hsp27 have emerged. OGX-427, the most successful representative of this group, has been shown to reduce the tumor size in prostate, pancreatic and lung cancer xenografts, particularly when combined with gemcitabine or erlotinib, as well as in mice models [213]. In clinical trials, OGX-427 decreased the expression of cancer markers and the amount of circulating tumor cells in ovarian and prostate cancer patients, and was well-tolerated [214]. After the OGX-427 phase II trial in castrate-resistant prostate cancer patients, over 70% have not experienced progression [215], but this agent did not improve the efficacy of the carboplatin–pemetrexed regimen towards metastatic non-small-cell lung cancer [216].

The third approach is the use of specific peptide aptamers to target Hsp27, developed due to the challenges posed by antisense technology in vivo. These short amino acid sequences are designed to bind specific domains of Hsp27 and disrupt its oligomerization, thereby impairing its functionality [217]. Similarly as in the case of direct inhibitors, the use of aptamers provides higher efficiency in combination with other anticancer drugs than with the application of the aptamer alone [218]. Aptamers known as PA11 and PA50 were shown to have anticancer efficacy in cell cultures and mouse xenograft models, but their potential clinical use is restricted by several limitations [213]. In Section 3.1.3, we emphasized the key role of Hsp27 in driving chemoresistance. Hence, the idea of targeting this chaperone emerged as a supportive approach in overcoming this issue, and indeed encouraging results were obtained in cellular models. The Hsp27-binding properties of BVDU have also been shown in cellular and animal models involved in the sensitization of drug-resistant cells to gemcitabine, cisplatin, cisplatin and cyclophosphamide, and, in some cases, the reversal of resistance. However, clinical studies involving BVDU alone or combined with gemcitabine did not entirely provide the expected results, since some pancreatic cancer patients developed toxic adverse effects despite the increase in the overall survival rate [208,218,219]. Heinrich et al. [206], using computational drug repositioning approach, indicated six compounds of diverse structures, including guanine derivatives, indomethacin, chlorpromazine—an antipsychotic known for its potent anticancer activity [220]—or its analog acepromazine, and have experimentally shown that these drugs present strong binding to Hsp27 and require lower doses to modulate its activity than previously known inhibitors, represented in this study by BVDU. Moreover, all of the tested compounds were shown to significantly decrease the resistance of lymphoma cells to an anticancer bortezomib [206]. In non-small-cell lung cancer, the Hsp27 antisense drug OGX-427 has been shown to sensitize cells to erlotinib and increase the anticancer efficacy of the combined therapy with the use of this drug, while Hsp27 itself was activated during the erlotinib treatment and protected the NSCLC cells from treatment-induced apoptosis [221]. Besides that, proteomic studies have demonstrated that exposure to resveratrol leads to the inhibition of Hsp27 expression in MCF7 breast cancer cells and sensitizes them to DOX treatment, manifested by the significant increase in the apoptosis rate in comparison to the control cells [222]. At present, attempts to design novel inhibitors targeting oncogenic Hsp27 are still ongoing, with the use of computer-assisted drug discovery and design, in order to develop effective compounds with a low toxicity that are potentially useful in cancer treatment and in overcoming chemotherapy resistance [223].

#### 4.1.2. Hsp60-Aimed Therapies

In recent years, numerous agents have been tested in the context of Hsp60 inhibitory activity, among which were plant-derived molecules, accepted drugs and known bioactive compounds [224]. The reduction in Hsp60 activity in tumor cells with the use of these compounds can rely on the direct binding and subsequent inhibition of this chaperone or affecting the regulation of Hsp60 expression, as well as its post-translational modifications. The first of the two concepts utilized in the design of the Hsp60 functional inhibitors is based on targeting sites crucial for ATP binding and hydrolysis, while, in the second concept, the cysteine residues of Hsp60 are targeted, either to be oxidized or form covalent bonds with the electrophilic moieties of a drug candidate molecule [225,226]. A well-known example of an Hsp60 inhibitor is mizoribine—an imidazole nucleoside antibiotic with immunosuppressive properties, shown to bind Hsp60 and impair its ATPase activity, thereby reducing the rate of protein folding [227]. However, the inhibitory concentration of mizoribine greatly exceeds the achievable concentration of this agent in plasma, hence some chemical optimization of this drug is needed to enable its clinical application [126].

Mitochondrial Hsp60 has been demonstrated to be a target for myrtucommulone A, a plant-derived compound which inhibits the refolding capacity of the Hsp60/Hsp10 complex [228]. Owing to the fact that myrtucommulone has other biological targets and, in addition to Hsp60, affects the arachidonic acid metabolism, this compound can provide a starting point for the development of analogs deprived of these activities [229]. Epolactaene, found in the fungal strain Penicillium sp., is another natural product reported to selectively inhibit Hsp60 activity through covalent binding to cysteine residues close to the ATP binding pocket, which indicates the allosteric regulation of Hsp60 without affecting its ATPase activity [230]. The folding activity of the Hsp60/Hsp10 complex can also be disturbed by curcumin in a dose-dependent manner, as reported in neuroblastoma cells [231].

Some classical anticancer agents have been found to influence Hsp60 expression. Exposure of human lung mucoepidermoid carcinoma cells to DOX has been shown to induce Hsp60 lysine acetylation, impairing the formation of the Hsp60/p53 complex related to cancer progression, and thereby increasing the levels of free p53, which in turn activates the tumor-suppressive cell senescence pathway [232]. Geldanamycin, a chemotherapeutic effective against osteosarcoma cells, acts in a similar manner, since it upregulates Hsp60 gene expression and induces its hyperacetylation via activating the HSF1 transcription factor, as shown on a corresponding cell line. The simultaneous reduction in the mitochondrial pool of Hsp60 augments apoptosis in cancer cells [233]. Overall, this indicates that inhibiting Hsp60 can contribute to the mitigation of drug resistance and improve the response to chemotherapy [234].

#### 4.1.3. Hsp70–Hsp40 and Hsp70–Hsp110 Axis

Hsp70 can be considered a druggable target due to the possibility of directly inhibiting its ATPase activity via interacting with the N-terminal ATP binding domain. However, targeting ATP binding has emerged as challenging due to the high affinity of ATP and ADP to Hsp70, and the success in this field remains limited, since a number of reported Hsp70 inhibitors do not have clearly elucidated mechanisms of action and in vitro biochemical evidence [235]. The classification of Hsp70 inhibitors is based on their mechanism of action to those targeting NBD, the C-terminal peptide-binding domain (SBD) or Hsp70 co-chaperones [236]. The first group is based on adenosine-related compounds that fit the ATPase domain of Hsp70 family members, selectively inhibiting their chaperone activity. As an example, the compound VER-155008 has been shown to induce caspase-dependent apoptosis in breast cancer cells and caspase-independent apoptosis in colon cancer cells [237]. As a commonly used method, high-throughput screening has been used to identify azure C, methylene blue and myricetin as Hsp70 inhibitors, but with undefined specificity towards inducible Hsp70s [238]. Moreover, an apoptosis-inducing imidazole derivative, apoptozole, as well as some sulfoglycolipids, dihydropyrimidines and peptide aptamers have the ability to inhibit Hsp70 ATPase activity in vitro at a specific stage of the ATPase cycle [148,236].

On the other hand, SBD inhibitors are developed to prevent protein–protein interactions between Hsp70 and its substrates, thereby blocking the cancer-related molecular mechanisms of Hsp70 activity [239]. Among them, the most representative one is Pifithrin-μ—a compound with inhibitory properties on the p53 transport to the mitochondria, and which is, moreover, selectively cytotoxic towards a variety of tumors, but not healthy cells, in a caspase-independent mechanism based on the aggregation of misfolded proteins and impaired lysosomal, as well as proteasomal, system function. The interaction of Pifithrin-μ with inducible Hsp70 causes the disruption in its association with Hsp40 and a wide array of client proteins, including key proteins involved in apoptosis, tumor suppressor p53 or autophagy-related p62 [240,241]. Pifithrin-μ has also been observed as being efficient against non-small-cell lung cancer, acute leukemias, prostate cancer and CRC [242,243]. Moreover, in three different leukemia cell lines, Pifithrin-μ enhanced the cytotoxicity of anticancer agents such as cytarabine and sorafenib, suggesting its potential therapeutic role in combination with other antineoplastics [243].

Since Hsp70 is closely functionally related to the family of smaller heat shock proteins, Hsp40s, which are required to perform its ATPase activity, the modulation of these co-chaperone functions highly affect the Hsp70 protein network, its integrity and mutual interaction. Plenty of synthetic compounds have been tested for anti-Hsp40 activity, and their number is still growing [244]. Particularly, farnesyl transferase inhibitors are studied as factors that could be used in combination treatment regimens. Some of these expectations are laid in KNK437, a benzylidene lactam compound, which inhibits the synthesis of Hsp40 family members and with a minor effect on the levels of Hsp27, 90 and 70 in colon cancer cell lines, indicating its potential in colorectal cancer (CRC) treatment [8,105]. KNK437 limits the growth and metastasis of colorectal cancer cells by interacting with the DNAJA1-cell division cycle protein 45 (CDC45) axis, but the detailed mechanism remains unknown. In mice, the combined administration of KNK437 and 5-FU/oxaliplatin was found to have a much more effective therapeutic outcome on CRC liver metastasis than monotherapy [245]. Cabazitaxel and tipifarnib have been found to be suppressive towards Hsp40s in prostate cancer and glioblastoma, and the latter acted as a farnesyltransferase inhibitor [244]. Among the known but not cytotoxic drugs, atorvastatin—an HMG-coenzyme A reductase—has been found to exert an anticancer effect on pancreatic carcinoma cells by suppressing Hsp40 member A1 (DNAJA1) farnesylation, resulting in other effects, such as mutant p53 degradation, p21 induction and followed by the increase in the apoptosis rate [246].

In the context of lung cancer therapy, the modulation of the Hsp40 family member B1 has profound implications. In examined A549 cells, its inhibition was correlated with the increased rate of gefitinib-induced apoptosis of malignant cells, suggesting that stabilizing mitogen-inducible gene 6 (MIG6) by suppressing DNAJB1 levels could increase the sensitivity of lung cancer cells to EGFR-targeting tyrosine kinase inhibitors like gefitinib [247]. Thus, targeting DNAJB1 could improve the therapeutic efficacy against gefitinib-resistant lung cancers, marking a possible advance in cancer treatment methods.

According to the aforementioned report in Section 3.2.3, detailing that the Hsp40 family member B8 is involved in resistance to docetaxel [114], it seems likely that its inhibition could be used as a therapeutic factor able to sensitize renal cancer cells to this anticancer agent, which indicates a need for studies on its practical application.

#### 4.1.4. Hsp90 Strategies

Since the inhibition of the Hsp90 chaperone cycle can lead to the blockage of multiple oncogenic signaling pathways, blocking the crucial changes upon which cancer cells depend for their growth and survival, the application of Hsp90-interfering agents emerges as a promising therapeutic approach with pleiotropic effects on proliferating cells [183,248]. Two types of Hsp90 inhibitors are distinguished, CTD- or NBD-targeting factors. Binding to CTD prevents the interaction with oncogenic client proteins and peptides, inducing their degradation, but none of proposed compounds have managed to enter clinical trials yet. NBD inhibitors are more studied, with the leading compounds classified as geldanamycin derivatives, which are able to block the phosphate region of Hsp90’s ATP-binding pocket. These compounds exert anticancer efficacy in vitro in a broad range of cancer types, and also in the following clinical trials, but only with moderate effects while used in monotherapy [17]. Moreover, in the case of geldanamycin, the emergence of adverse effects such as hepatotoxicity and drug resistance induction was observed in clinical trials [178]. Another limitation, making clinical use of Hsp90 inhibitors controversial, is the fact that their application entails an increased synthesis of Hsp70 as a compensatory mechanism in cells, which indicates the potential necessity to combine them with Hsp70 inhibitors [236].

A valuable finding from the studies conducted to date is that the combination of Hsp90 inhibitors with some conventional anticancer agents, e.g., platinum compounds, imatinib, trastuzumab, etoposide, DOX or histone deacetylase inhibitors, has additive or synergistic effects on cancer cells [249]. Efforts have also been made in order to selectively target and inhibit the organelle-specific Hsp90 chaperone function [250]. In addition to client proteins, the interactions of Hsp90 with its co-chaperones can be disturbed by Hsp90 inhibitors [251]. Currently, the most promising outcomes as assessed in preclinical trials are provided by ganetespib, a second-generation Hsp90 inhibitor containing a resorcinol moiety of radicicol derivatives, which has shown strong cytotoxic effects towards several cell lines representing various types of solid cancers (e.g., breast, lung, prostate and gastric cancers), as well as hematological cancers such as acute myeloid leukemia. Its mechanism of action is based on degrading oncogenic signal transduction proteins, which are Hsp90 clients, thus inhibiting cancer progression. Importantly, ganetespib is characterized by a better safety profile, since it seems to cause fewer adverse effects than other Hsp90 inhibitors, and is currently being tested in a phase II clinical trial in combination with fulvestrant as a new therapeutic strategy for hormone receptor-positive metastatic breast cancer [252]. 

Another strategy besides the direct inhibition of Hsp90 is decreasing its level, e.g., with the use of novel proteolysis-targeting chimeras, which are able to cause particular protein degradation through cellular ubiquitin systems [253]. Also, the design of antibodies against Hsp90 present in the extracellular milieu as a cell membrane-bounded protein seems to be of high potential, since Hsp90 is expressed in greater amounts on cancer cell surfaces compared to healthy cells. Hyun et al. [254] succeeded in developing an antibody–drug conjugate against Hsp90 which reduced the cell viability and colony formation of non-small-cell lung cancer to a significant extent. The Hsp90 peptide, along with the melanoma-derived peptide, are components of an anticancer vaccine, known under the name Oncophage, which has been approved in early-stage kidney cancer therapy [255].

#### 4.1.5. Hsp110

As Hsp110 participates in many inter-related processes ensuring cell survival, such as the maintenance of lysosomal membrane integrity, STAT3 activation or DNA repair, and owing to its significant role in CRC development, its targeting seems to be attractive in new therapeutic strategies towards this type of malignancy. However, the findings in this field are so far limited. Seemingly, the most significant achievement to date is the identification of two chemical inhibitors which interfere with STAT3 binding by Hsp110, thereby reducing the cancer cell proliferation rate and inhibiting tumor growth [195]. Due to the structural similarity, some Hsp110 inhibitors may also influence Hsp70, which can result in enhanced efficacy [256]. Another example of the therapeutic application of Hsp110 is the development of a complex vaccine where recombinant human Hsp110 is one of the constituents, which is reported to induce an anticancer response in advanced-stage melanoma patients in a small clinical study [257].

### 4.2. Heat Shock Proteins in Cancer Immunotherapies

It is a well-known fact that immune cells inhabiting the tumor microenvironment, such as T-cells, DCs or macrophages, contribute to the formation of immunosuppressive conditions by releasing cytokines, which induce macrophage M2 phenotype acquisition, regulatory T-cell activation and myeloid-derived suppressor cells (MDSCs) to gather at the tumor site. Collectively, cancer immune tolerance develops, and the activity of the aforementioned immune cells increases the aggressiveness and metastatic ability of the tumor cells. An approach to overcome such tolerance is immunotherapy, triggering both the innate and adaptive immune responses directed towards the cancer cells. Although the Hsp family members are in general cytoprotective, some specific features of particular Hsps can be employed to design novel immunotherapeutic regimens [258]. As mentioned before, Hsps are localized in specific cellular compartments, but can also be released into the extracellular milieu as a result of the necrotic disintegration of cells, in extracellular vesicles (EVs) or can remain as membrane-associated. Both extracellular and intracellular Hsps have been shown by numerous studies to play a significant role in the regulation of immune responses, and their reported functions include both immunostimulatory and immunosuppressive properties, dependent on the cellular context of their release [259]. Concerning their immunostimulatory properties, which are attracting attention due to their high potential to be exploited clinically, Hsps such as Hsp90 can promote the cross-presentation of antigens and Hsp-associated peptides to the major histocompatibility complex class I and class II (MHC-I/II), enhancing, respectively, CD8+ cytotoxic and CD4+ helper T lymphocyte activity [205]. This occurs due to the capture of the complexes formed by the Hsp and peptide antigens by the antigen-presenting cells (APCs), like dendritic cells or macrophages, via nonspecific receptor-mediated endocytosis. The major receptors mediating this process were identified as scavenger receptors, class E member oxidized low-density lipoprotein receptor-1 (LOX-1) and class F member 1 (SCARF1), also known as scavenger receptor expressed by endothelial cells-1 (SREC1) [260]. The internalization of the Hsp–antigenic peptide complex through endocytosis can also be promoted by extracellular Hsp90 [261], and has been shown to be more efficient in comparison to the internalization of only soluble antigens. In addition, the interaction between Hsp and APC positively affects the maturation of APCs through the upregulation of costimulatory molecules and cytokine secretion [262]. Moreover, Hsp70 increases the cytolytic properties of NK cells, accelerates dendritic cell maturation and induces the secretion of cytokines, chemokines or small-molecular-weight mediators such as prostaglandins [261,263]. The latter occurs as a result of NF-kB activation via Hsp70 binding to monocytes, inducing intracellular calcium flux; thereby, Hsps are referred to as “chaperokines”, participating in signal transduction [264,265]. The mounting evidence supports the finding that Hsp70 activates innate immune responses via TLR signaling in vivo [266]. The immunomodulatory capacity was also reported for Hsp60, which, after purification from the mouse sarcoma, along with Hsp70 and Hsp110, triggered an anticancer immune response in autologous tumors [267].

Since some cancer cells express only a few neoantigens, i.e., proteins absent in healthy tissues and formed due to tumor-specific alterations on the tumor cell surface, such cross-presentation of Hsp–peptide complexes expands the spectrum of available immune system targets [205]. Hence, this phenomenon is crucial in cancer immunosurveillance. Hsp70 is also known to be capable of inducing both innate and adaptive immune responses directed towards tumor cells, either on its own or when associated with immunogenic peptides. Similarly to Hsp90, Hsp70 enhances antigen uptake, processing and presentation, stimulating T-cell activation. Additionally, membrane-bound Hsp70 can be considered a tumor-specific antigen, which can be recognized by previously preactivated NK cells and killed via granzyme B-mediated apoptosis [268]. Hsp110, due to its strong chaperone capability, has an important role in antigen presentation as well, and its recombinant form has been used to design natural chaperone and large protein antigen complexes as cancer vaccines, which were shown to trigger a strong T lymphocyte response in preclinical models [269,270]. Likewise, complexes composed of Hsp110 and the intracellular domain of HER-2/neu induced specific CD8+ and CD4+ T-cell responses, which, in mice models, have considerably limited cancer development [271]. This all indicates that immunogenic peptides chaperoned by Hsps, due to their ability to elicit specific immune response against cancer cells, can be a vaccination target in anticancer therapies [263], whereas some Hsps might be used as vaccine adjuvants due to their intrinsic stimulatory properties [272].

Hsp–peptide complexes present in the cells of each tumor are characterized by an individual antigenic and neoantigenic pattern, a peptide “fingerprint”, unique for specific cancer which can be used in personalized therapy approaches, where the surgical resection of the tumor, followed by the isolation of the complexes, is a basis for anticancer vaccine production. This kind of procedure would enable the tailoring of immunotherapy to each patient based on autologous products instead of selecting subgroups of patients fitting to the drug. However, this entails risks concerning safety and efficacy issues [273]. Another limitation concerns the poor access to some tumors, as well as complex and laborious vaccine preparation protocols [272]. Anticancer vaccines based on Hsp70–peptide complexes, obtained from autologous tumor lysates, have been introduced into clinical trials of various cancers, including melanoma, glioblastoma, renal, gastric or pancreatic carcinoma, and, although in a significant number of patients the immune responses were induced, only in certain subgroups was this effect clinically relevant [263]. Hsp70–peptide complexes are also prepared with the use of tumor–DC fusion cells, and this approach allows for the additional optimization of antigen processing and peptide loading, producing most promising outcomes [274,275]. Yu et al. [272] prepared a recombinant large chaperone–protein complex vaccine with a broad reservoir of potential peptides which could be used as a synthetic platform serving as a multivalent vaccine towards various antigen targets. The authors suggest that the implementation of the pathogen-associated molecular patterns, with a key role in the recognition and inducing of innate immune response, as the “danger signal” to the recombinant vaccine system, would lead to improved treatment efficiency and allow for the mitigation of immunosuppressive conditions in the tumor microenvironment.

A more recent type of cancer immunotherapy, the immune checkpoint blockade, has recently gained considerable attention due to the remarkable clinical effects observed in some patients [276]. These negative feedback mechanisms of the immune system, generally preventing excessive immune responses, are often reprogrammed and used by cancer cells to escape immune surveillance [273]. Hsps have also been shown to be implicated in the modulation of immune checkpoints. In this regard, the role of Hsp90 seems to be particularly important, since it has been demonstrated to modulate the expression of programmed cell death ligand 1 (PD-L1) and 2 (PD-L2) on the surface of cancer cells and macrophages via STAT3 activation, and the upregulation of the c-Myc oncogene, enabling cancer cells to avoid T-cell-mediated cytotoxicity [277]. Moreover, the c-Myc protein and nucleophosmin/anaplastic lymphoma kinase (NPM/ALK), responsible for STAT3 activation in T-cell lymphoma, are client proteins for Hsp90 [278]. Studies on Hsp90 inhibitors have also shown that this protein is implicated in CTLA4 immune checkpoint regulation, and thus has been suggested that the complementary targeting and suppression of Hsp90 activity would be beneficial and improve the efficacy of cancer immunotherapy [279]. Also, according to Proia et al. [280], the inactivation of Hsp90 can be beneficial in the context of sensitizing cancer cells to cytotoxic agents while combined with immune checkpoint inhibitors in order to boost the intrinsic immune response in tumor cells. On the other hand, Bae et al. [281] reported that inhibiting Hsp90 exerts a considerable effect on the functionality of T lymphocytes and NK cells, since it irreversibly reduces the expression of critical antigens and molecules on their surface, which disrupts the activation, proliferation and section of IFN-γ by these cells. Moreover, it is accompanied by decreased cytotoxicity towards cancer cells, which has important clinical implications, since Hsp90 inhibition can lead to immune suppression.

## 5. Conclusions

Hsps play an important role in cellular homeostasis and protection against various stressors, including those associated with cancer. Rapid proliferation, genomic instability, metabolic alterations and oxygen/nutrient deprivation constitute a challenging environment where cancer cells, in order to survive and multiply, employ various mechanisms, and the overexpression of Hsps is crucial to maintain the stability and functionality of oncoproteins. Hsps, as thoroughly described in this review work, are associated with plenty of inter-related signal transduction pathways and signaling hubs essential for tumor survival and cell death inhibition, including those contributing to chemotherapy resistance development. Hence, understanding the complex interplay between Hsps and cancer is an actively pursued area of research—Hsp-based approaches are considered to have a great contribution to oncoimmunology progress and to support the development of anticancer therapeutic regimens of higher efficacy and lower systemic toxicity.

Direct inhibitors of Hsps are continuously developed and optimized, as well as combination therapies that include Hsp-targeting factors, along with traditional chemotherapy or immunotherapy. Definitely, some advancements are required in the evaluation of the safety and efficacy of such approaches in different cancer types and stages. Gathering more evidence from clinical trials will be crucial for determining the full therapeutic potential of Hsp-related treatments. The potential employment of Hsp-based therapies might also involve the need to identify patient populations or cancer types that would be more responsive to such treatment, which is in line with the personalized medicine approach. However, in order to reach these goals, further studies at the molecular level enabling a broader understanding of Hsp functionality in cancer cell biology and the modulation of the tumor microenvironment are needed. Understanding the Hsp expression profiles in individual tumors may help guide therapeutic decisions, while the validation of some Hsp-related biomarkers could assist in the treatment selection of cancer patients. Additionally, functional genomics approaches would be supportive in unraveling the detailed and specific roles of individual Hsps in different cancers, which could guide the development of more targeted and selective therapies.

## Figures and Tables

**Table 1:** A summary of heat shock proteins found to be associated with resistance to specific chemotherapeutic agents, confirmed by research reports and presented with respect to the cell lines on which the referred studies were conducted.

	Chemotherapeutic	Cancer Type (Cell Line Studied)	Ref.
Hsp27	doxorubicin	breast cancer (MCF7, MDA-MB-231, MDA-MB-435) colon cancer (CaCo2, HT-29), prostate cancer (LNCaP)	[58]
	trastuzumab	breast cancer (SK-BR-3)	[83]
	gemcitabine	pancreatic cancer (SW1990)	[85]
	5-fluorouracil	colon cancer (HT-29)	[93]
		hepatocellular carcinoma (Hep3B, HepG2)	[94]
Hsp40	docetaxel	renal cell carcinoma (RenCa)	[114]
	5-fluorouracil	hepatocellular carcinoma (Hep3B, HepG2)	[94]
Hsp60	cisplatin, oxaliplatin	ovarian cancer (A2780), bladder cancer (UCRU-BL13)	[137]
	cisplatin	head and neck cancer (UMSCC5, UMSCC10b)	[138]
		cervical cancer (HeLa)	[140]
	tamoxifen	breast cancer (MCF-7)	[141]
	5-fluorouracil	colorectal cancer (SW480)	[143]
Hsp70	imatinib	chronic myeloid leukemia (K562)	[152]
	ibrutinib	chronic lymphocytic leukemia (patient-derived samples)	[154]
	gemcitabine	pancreatic ductal adenocarcinoma (human or mouse tissue samples)	[155]
	gemcitabine, topotecan	fibrosarcoma (WEHI)	[157]
	5-fluorouracil	colon cancer (HT-29, SNU-C4)	[158]
	paclitaxel	chronic myeloid leukemia (K562), acute promyelocytic leukemia (HL-60), patients’ bone marrow aspirates	[160]
	cisplatin	gastric cancer (HGC-27)	[164]
		ovarian cancer (OV2008, A2780)	[163]
Hsp90	paclitaxel, cisplatin	ovarian cancer (A2780)	[181]
	5-fluorouracil, gemcitabine	pancreatic ductal adenocarcinoma (HPAC, PANC-1)	[183]
	melphalan	multiple myeloma (RPMI8226)	[184]
Hsp110	doxorubicin	cervical cancer (HeLa)	[204]
	oxaliplatin	colorectal cancer (SW480)	[197]

## Data Availability

The data presented in this study are available on request from the corresponding author.
